# The Effect of Sugarcane Straw Aging in the Field on Cell Wall Composition

**DOI:** 10.3389/fpls.2021.652168

**Published:** 2021-07-15

**Authors:** Débora Pagliuso, Adriana Grandis, Cristiane Ribeiro de Sousa, Amanda Pereira de Souza, Carlos Driemeier, Marcos S. Buckeridge

**Affiliations:** ^1^Laboratory of Plant Physiological Ecology, Department of Botany, Institute of Biosciences, University of São Paulo, São Paulo, Brazil; ^2^Carl R. Woese Institute for Genomic Biology, University of Illinois at Urbana-Champaign, Urbana, IL, United States; ^3^Brazilian Biorenewables National Laboratory, Brazilian Center for Research in Energy and Materials, Campinas, Brazil

**Keywords:** sugarcane straw, cell wall, decomposition, recalcitrance, polysaccharide

## Abstract

Cellulosic ethanol is an alternative for increasing the amount of bioethanol production in the world. In Brazil, sugarcane leads the bioethanol production, and to improve its yield, besides bagasse, sugarcane straw is a possible feedstock. However, the process that leads to cell wall disassembly under field conditions is unknown, and understanding how this happens can improve sugarcane biorefinery and soil quality. In the present work, we aimed at studying how sugarcane straw is degraded in the field after 3, 6, 9, and 12 months. Non-structural and structural carbohydrates, lignin content, ash, and cellulose crystallinity were analyzed. The cell wall composition was determined by cell wall fractionation and determination of monosaccharide composition. Non-structural carbohydrates degraded quickly during the first 3 months in the field. Pectins and lignin remained in the plant waste for up to 12 months, while the hemicelluloses and cellulose decreased 7.4 and 12.4%, respectively. Changes in monosaccharide compositions indicated solubilization of arabinoxylan (xylose and arabinose) and β-glucans (β-1,3 1,4 glucan; after 3 months) followed by degradation of cellulose (after 6 months). Despite cellulose reduction, the xylose:glucose ratio increased, suggesting that glucose is consumed faster than xylose. The degradation and solubilization of the cell wall polysaccharides concomitantly increased the level of compounds related to recalcitrance, which led to a reduction in saccharification and an increase in minerals and ash contents. Cellulose crystallinity changed little, with evidence of silica at the latter stages, indicating mineralization of the material. Our data suggest that for better soil mineralization, sugarcane straw must stay in the field for over 1 year. Alternatively, for bioenergy purposes, straw should be used in less than 3 months.

## Introduction

New energy resources for a sustainable environment and reduction of oil use has been growing. Among the diverse feedstocks, sugarcane has enormous potential for bioethanol and bioelectricity production. Concerning bioethanol, both first-generation (1G) and second-generation (2G) can be employed using sucrose and biomass from sugarcane, respectively, (de Souza et al., 2014). Brazil is the largest sugarcane producer globally, being responsible for 40% of global production (Cherubin et al., 2019; Ullah et al., 2020). Sugarcane in Brazil has been economically important since the 16th century, but only in the 30s, it was introduced as a fuel source due to energy security (Cortez and Baldassin, 2016). The oil crisis of the 70s led to the launch of the PRO-ÁLCOOL program, which led Brazil to establish the 1G technology so that ethanol as a biofuel became nationalized (de Souza et al., 2014). The cultivated area in the 2019/2020 harvest corresponded to 8.5 million ha with an estimative of 642.7 Mt of millable cane (Conab, 2020), and 55% of the planted area belongs to the state of São Paulo (Rudorff et al., 2010) along with 48.5% of the ethanol production (Conab, 2020).

Sugarcane production changes according to the crop variety, edaphoclimatic conditions, and management practices (Carvalho et al., 2013). To facilitate the harvest, it was common to burn sugarcane residues (Leal et al., 2013), with practically all being manually harvested. However, due to the Agro-Environmental Protocol proposed by the Sugarcane Industry Association (UNICA – União da Indústria de Cana-de-Açúcar) and the government of the state of São Paulo, the burning practice was prohibited. Therefore, legislation was introduced to impose mechanized harvesting, eliminating sugarcane burning and leaving a thick layer of straw on the soil surface (10 to 20 Mg ha. year^–1^; Menandro et al., 2017; Santoro et al., 2017; Carvalho et al., 2019). The straw left on the soil surface is usually maintained for 2 weeks to decrease its water content by natural drying in the bailing system (Cardoso et al., 2013). Then straw is withdrawn, baled, and transported to the industry (Hassuani et al., 2005; Cardoso et al., 2013; Lisboa et al., 2018). This practice sustainably produces sugarcane, increasing profits with the straw management to new products such as bioethanol, bioelectricity, or bio-renewable feedstocks (Hassuani et al., 2005; Cardoso et al., 2013; Lisboa et al., 2017, 2018; Carvalho et al., 2019).

Sugarcane straw (also known as trash) is composed of 54% dry leaves and 46% green tops with a moisture of 30–60% after the harvest (Paes and Oliveira, 2005; Michelazzo and Braunbeck, 2008; Carvalho et al., 2013; Lisboa et al., 2018). This feedstock holds 1/3 of the total energy available in the plant in the form of the cell wall polysaccharides (de Souza et al., 2014). The maintenance of the straw on the field is essential for carbon accumulation, nutrient recycling, water storage and infiltration, protection against erosion, soil temperature and bulk density, and biological activity (Cerri et al., 2011; Fortes et al., 2013; Paredes et al., 2015; Ng Cheong and Teeluck, 2016; Valim et al., 2016; Galdos et al., 2017; Nxumalo et al., 2017; Lisboa et al., 2018).

Alternatively, the energy potential of sugarcane straw is concentrated in the cell wall polysaccharides (carbohydrates). Thus, it is valuable material for 2G bioethanol production. In this regard, it is crucial to know their composition and structural features. The plant cell wall is a complex structure formed by a cellulose core surrounded by hemicelluloses and lignin, all immersed in a pectin matrix (Carpita and Gibeaut, 1993). The cell walls are said to display a Glycomic Code, which holds enormous complexity behind the assembly of its polymers (Buckeridge and de Souza, 2014; Buckeridge, 2018). The Glycomic Code was proposed as a concept in which information is held in the carbohydrates of the extracellular matrices (Buckeridge, 2018). Thus, the structure’s disassembly is still considered challenging due to the interactions among the polysaccharides and the composition diversity among tissue and species. In sugarcane, pectins and β-glucan (hemicellulose) are more accessible to enzyme hydrolysis in 2G processes. Alternatively, the hemicelluloses arabinoxylan and xyloglucan are more closely attached to cellulose, conferring some degree of recalcitrance to the structure. In 2013 De Souza et al. proposed a model for the hydrolysis of sugarcane biomass in which polymers would be selectively and sequentially attacked by hydrolases. The attack starts on the pectins, progressing toward the more soluble hemicelluloses. After it hydrolyses the hemicelluloses more closely attached to cellulose, it ends up exposing cellulose microfibrils to the attack of cellulases. This model was partly confirmed to function for microorganisms (Borin et al., 2015) and *in vivo* in sugarcane tissues (Leite et al., 2017; Grandis et al., 2019).

Straw is a fibrous and heterogeneous material with a reported chemical composition of 19–43% lignin, 29–44% cellulose, 27–31% hemicelluloses, and 2.4–7.8% ash (Costa et al., 2013; de Barros et al., 2013; Landel et al., 2013; Oliveira Moutta et al., 2013; Oliveira et al., 2014; Szczerbowski et al., 2014). However, in many studies, pectins are disregarded, whereas in grasses (including sugarcane), these polymers have been reported as being 10% of the cell wall (Carpita, 1996; de Souza et al., 2013, 2015). Pectins are relevant because they interfere with the wall’s permeability and therefore mediate the access of hydrolases to cellulose and hemicelluloses (Bellincampi et al., 2014; Latarullo et al., 2016; Grandis et al., 2019). Likewise, a fine characterization of the straw polysaccharides and their disassembly during field degradation, together with the interactions of biotic and abiotic factors, may improve the use of the straw for biorefinery and correlate with soil protection.

Here, we evaluated the cell wall degradation process of sugarcane straw for 1 year. By following carbohydrate composition, lignin levels, uronic acids, cellulose crystallinity, and ash, this study was able to construct a view to understand the cell wall degradation and the raise of mineral impurities in biomass by the environmental and natural field conditions. As straw aged, non-structural carbohydrates were degraded whereas the structural carbohydrates started to be modified and partially consumed. While crystallinity of cellulose changed little and SiO_2_ (quartz) signal increased as a response to the biomass relative proportion alteration (a sign of increased mineralization), the proportion of lignin and pectins increased in biomass, provoking a decay in the saccharification capacity. Thus, for up to 3 months in the field, straw biomass could be either used directly for 2G bioethanol production (or bioelectricity) or left for more than 1 year to improve soil mineralization and further recovery. Combination of both strategies could also be suitable to improve sugarcane sustainability.

## Materials and Methods

### Plant Material and Site Description

**FIGURE 1:**
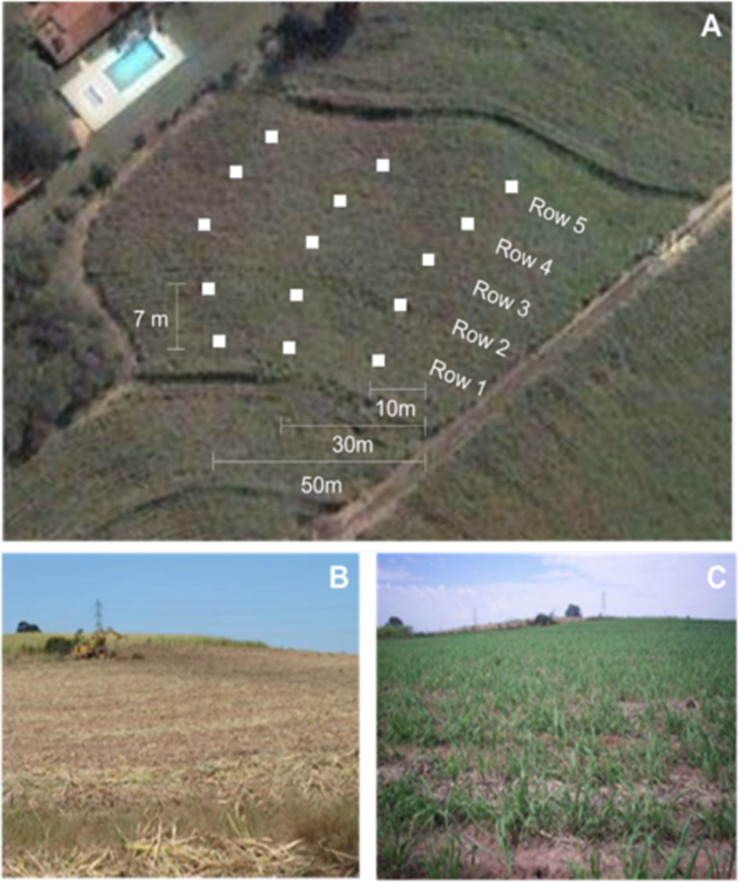
Representation of the sugarcane field studied in Piracicaba, São Paulo, Brazil (S 22° 43′07.9″, W 47°C 41′91.7″). **(A)** Experiment site area from a satellite view (Google maps). The field plot is shown in the center and the white squares represent the harvesting sites distribution. **(B)** Field of sugarcane cultivation after the harvest. **(C)** Field after 3 months with the new sugarcane plants sprouting.

The study was conducted in Piracicaba, São Paulo, Brazil (S 22° 43′07.9″, W 47°C 41′91.7″) in a sugarcane (*Saccharum spp.* cv. SP80-1816) field plot with an area of 10,000 m^2^. After the manual harvesting of sugarcane, the remaining straw was placed in 5 rows separated by 7 m from each other. Pooled samples were collected at three points, following a spacing of 10–20 m among the rows in a field plot (Figure 1A). Five harvests were performed every 3 months along the period from June 2009 to June 2010. The field condition on the first harvest and the regrowth of sugarcane after 3 months can be seen in Figures 1B,C, respectively. The soil was separated from the plant material in the laboratory by water flotation. The plant material was freeze-dried and pulverized in a ball mill for further analyses.

### Soil Relative Humidity and Climatic Parameters

The soil in contact with sugarcane straw was collected and stored in glass jars. Samples were dried at 60°C for relative soil humidity until constant dry mass. The humidity was determined gravimetrically.

Climatic parameters (air relative humidity, temperature, and rainfall) were obtained from the meteorological station of ESALQ/USP^1^ located at S 22°30′30″, W 47°38′00″.

### Soluble Sugar Quantification

Total soluble sugars were extracted six times from 10 mg pulverized dry mass samples with 1.5 mL of 80% ethanol at 80°C for 20 min. The Alcohol Insoluble Residue (AIR) was dried at 45°C overnight and subsequently used for starch evaluation. After each extraction, the supernatant was recovered by centrifugation. The supernatant pool was vacuum concentrated (ThermoScientific^®^ Savant SC 250 EXP) and resuspended in 1 mL of water and 1 mL of chloroform. The recovered water-soluble sugars (sucrose, fructose, glucose, and raffinose) were analyzed by High Performance Anion Exchange Chromatography with Pulsed Amperometric Detection (HPAEC-PAD) in a Dionex system (ICS 5,000) using a CarboPac PA1 column and eluted with 150 μM NaOH on an isocratic run of 27 min (Pagliuso et al., 2018).

### Starch Extraction and Quantification

Starch was measured according to do Amaral et al. (2007) and Arenque et al. (2014). AIR was treated with 120 U⋅ mL^–1^ of α-amylase (E.C. 3.2.1.1) of *Bacillus licheniformis* (Megazyme^®^ Inc., Australia) diluted in 10 mM MOPS buffer pH 6.5 at 75°C for 1 h. Incubation was followed by the addition of 30 U⋅ mL^–1^ of amyloglucosidase (E.C. 3.2.1.3) of *Aspergillus niger* (Megazyme^®^ Inc., Australia) diluted in 100 mM sodium acetate buffer pH 4.5 at 50°C for 1 h. For starch determination, 50 μL of each sample was added to a 250 μL of a mixture containing glucose oxidase (1,100 U⋅ mL^–1^), peroxidase (700 U⋅ mL^–1^), 4-aminoantipirin (290 μmol⋅ L^–1^), and 50 mM phenol at pH 7.5. The plates were incubated for 15 min at 30°C, and the absorbance was measured at 490 nm. The standard curve was performed with commercial glucose (Sigma^®^).

### Cell Wall Fractionation

The cell wall fractionation protocol was adapted from de Souza et al. (2013). The soluble sugars of 500 mg of pulverized straw sugarcane were extracted six times, with 35 mL of 80% ethanol at 80°C per 20 min. The remaining material, AIR, was dried at 60°C for 6 h and submitted to starch removal with two extractions of 35 mL DMSO 90% for 12 h each. De-starched AIR (cell walls) was extracted with 40 mL of sodium chlorite 3% (m/v) in acetic acid 0.4% (v/v) for 1 h at 80°C followed by the addition of 400 mg of sodium chlorite and 160 μL of acetic acid and reaction of 2 h at 80°C for lignin removal. The supernatant containing soluble polysaccharides was recovered. The sodium chlorite residue was extracted with 30 mL of 0.5% ammonium oxalate (pH 7.0) at 80°C for 1 h with continuous stirring for pectin solubilization. For hemicellulose solubilization, the supernatants were recovered, and the ammonium oxalate-extracted cell wall residue was sequentially subjected to two extractions with 30 mL of 0.1, 1, and 4 M sodium hydroxide containing 3 mg⋅ mL^–1^ sodium borohydride, at room temperature for 1 h each. The supernatants containing the hemicelluloses were neutralized with glacial acetic acid. After the extractions, the pellet was washed five times with distilled water, frozen, and freeze-dried. The acquired fractions were dialyzed to remove salts, frozen and lyophilized. The yields of the cell wall domains were obtained gravimetrically.

### Cellulose Determination

The cellulose was determined on the 4 M sodium hydroxide residue after an Udgegraff solution digestion [5% nitric acid (v/v) and 15% acetic acid (v/v)] for 90 min at 100°C. Hydrolysis of residue discerns the cellulose content that was measured gravimetric after several washes and freeze-drying.

### Acid Hydrolysis of the Cell Wall

The cell wall was obtained after the extraction of the soluble sugar and the fractionation process. 2 mg of the integral cell walls AIR and the cell wall fractions were hydrolyzed in a thermoblock with 1 mL of 2 M TFA (trifluoroacetic acid) at 100°C for 1 h with continuous stirring (750 rpm). Then, the supernatants of all the samples were vacuum-dried and resuspended in 1 mL of distilled water. For cellulose quantification, the sulfuric acid method was used to obtain free glucose to quantify materials reminiscence after the TFA hydrolysis (de Souza et al., 2013). The cellulose was hydrolyzed with 72% (30 min), 4% (1 h at 121°C; v/v) H_2_SO_4_. The glucose solution was taken to a pH between 6 and 8 by adding 50% (w/v) NaOH.

Monosaccharides were analyzed using HPAEC-PAD. The column used was a CarboPac SA10 column (ICS 5,000 system, Dionex-Thermo^®^). The sugars were eluted isocratically with 99.2% of water and 0.8% sodium hydroxide 200nM (1mL⋅min^–1^). The monosaccharide was detected by Pulsed Amperometric Detection using a post-column base containing 500 mM NaOH (0.5 mL⋅ min^–1^). Quantification was performed injecting known concnetrations of arabinose, galactose, glucose, xylose, fucose, rhamnose, and mannose and calculating standard curves (Pagliuso et al., 2018).

### Uronic Acid Determination

The uronic acids content was determined according to Filisetti-Cozzi and Carpita (1991). Five mg of each cell wall fraction were hydrolyzed on ice with 2 mL of sulfuric acid for 10 min under stirring (1,250 rpm). For hydrolysis, 1 mL of deionized water was added, and the procedure was repeated once more. This final reaction was diluted to 10 mL and centrifuged at 4,000 *g* for 10 min at room temperature. Four hundred μL were submitted to the colorimetric essay at 100°C for 20 min with 40 μL of 4 M sulfamic acid/potassium sulfamate solution (pH 1.6) and 2.4 mL of 75 mM sodium borate in sulfuric acid. The reactions were cooled on ice, and 80 μL of m-hydroxybiphenyl in 0.5% NaOH was added. The samples were vortexed for color development and read on a spectrophotometer at 525 nm. The uronic acid quantification was determined based on a D-galacturonic acid in a concentration range of 5–40 μL/400 μL.

### Saccharification

For digestibility evaluation, powdered straw sugarcane was saccharified as described by Gomez et al. (2010). 4 mg of samples were treated with 0.5 N NaOH at 90°C for 30 min before enzymatic hydrolysis, which was conducted with an enzyme blend with 4:1 cellulase (*Trichoderma reesei*) and Novozymes^®^ 188 (cellobiase from *A. niger*; both Novozymes, Bagsvaerd, Denmark) at 30°C in 25 mM sodium acetate buffer at pH 4.5 for 18 h. Reducing sugars determination was carried out with 75 μL of the hydrolysis’ supernatant by MBTH colorimetric method. 25 μL of 1 M NaOH plus 50 μL of a solution containing 0.43 mg⋅ mL^–1^ MBTH and 0.14 mg⋅ mL^–1^ DTT were added to the hydrolyzate and heated at 60°C for 20 min. Then, 100 μL of oxidizing reagent [0,2% FeNH_4_ (SO_4_)], 0.2% sulfamic acid, and 0.1% HCl for color development. The standard curve for sugar determination had 50, 100, and 150 nmol of glucose (Sigma^®^). With a total of 250 μL in each well of the plate, reactions were revealed at 620 nm. This procedure was made at the York University and the authors thank Dr. Leonardo Gomez for the help.

### Lignin Extraction and Determination

Thirty milligrams of pulverized straw were washed with 1 mL of water, ethanol, ethanol-chloroform (1:1 v/v), and acetone in termoblock for 15 min at 98, 76, 59, and 54°C, respectively, under constant stirring of 750 rpm (van Acker et al., 2013). The reminiscent material was recovered by centrifugation for 5 min at 14,000 *g* and dried at 45°C overnight. Recently acetyl bromide method was pointed out as precise for grasses and forage (Fukushima et al., 2021). In 10 mg of dried washed material was added 250 μL of 25% acetyl bromide in acetic acid and incubated for 2 h at 50°C and 1 h with stirring of 1,500 rpm (Fukushima and Kerley, 2011; Fukushima et al., 2015). The samples were cooled at 4°C and centrifuge for 10 min at 14,000 *g*. The final reaction was conducted with 400 μL of 2 M sodium hydroxide, 75 μL of 0.5 M hydroxylamine hydrochloride, 1,425 μL glacial acetic acid, and 100 μL of acetyl-bromide supernatant solution and read at 280 nm. The lignin measurement was calculated by Bouguer-Lambert-Beer law (Eq. 1) and corrected by the cell wall amount used on the assay.

(1) A=ε*c*l

Where, ε = 23.35 l⋅ g^–1^⋅ cm^–1^ (Xue et al., 2008) and l = 0.1 cm.

### β-Glucan Hydrolysis

The polysaccharide β-glucan was analyzed by enzymatic hydrolysis with 0.5 U⋅mL^–1^ lichenase from *Bacillus subtilis* (Megazyme^®^, Australia) in 50 mM sodium acetate buffer at pH 5 for 24 hr at 30°C with 1 mg of the cell wall fractions. Commercial barley β-glucan (Megazyme^®^, Australia) was used as standard. The reactions were stopped by heating at 100°C for 5 min. The oligosaccharides fine-structure were analyzed by HPAEC-PAD using a CarboPac PA-100 column (ICS 3,000 system, Dionex-Thermo^®^). The column was eluted with 88 mM NaOH (baseline) and sodium acetate 200 mM NaOH (0.9 mL⋅ min^–1^) for 45 min. The peak areas from the tri- and tetrasaccharide nominated in the chromatograms as a and b shown in Figure 4 were used to calculate the tri:tetrasaccharides ratio.

### Carbon and Nitrogen Quantification

Dry samples were weighed (1.3–1.5 mg) and placed in plater capsules for combustion and volatilization. The volatile compounds were analyzed by mass spectrometry (Finnegan Delta Plus) for elemental C and N (Carlo-Erba, 1110). Sugarcane leaves with a known concentration of carbon and nitrogen were used as standards. The carbon and nitrogen concentration were expressed in percentage, and δ^13^Cor δ^14^N represented by the thousand (‰) concerning the standard. The isotopic calculation was performed according to the Eq. (2) in which *R* represents the isotopic ratio ^13^C/^12^C or ^15^N/^14^N of the sample and the standard. All the samples were analyzed in duplicates, accepting a maximum analytical error deviation of 0.3‰ for ^13^C and 0.5 ‰ for ^15^N. These analyses were performed at the laboratory of Prof. Plínio Camargo, CENA-USP, in Piracicaba, São Paulo.

$$\delta^{13}\,\text{C or}\,\delta^{14}\,\text{N}=\left(\frac{R_{\text{sample}}-R_{\text{standard}}}{R_{\text{standard}}}\right)*1000\,\left(I\,I\right)$$

### Biomass Ash Content

Ash contents were determined gravimetrically by calcination (Sluiter et al., 2008).

### Cellulose Crystallinity

X-ray diffraction was performed with air-dried ground biomass inserted in capillary tubes. The tubes were illuminated orthogonally by Cu Kα (λ = 1.5418 Å) radiation from a rotating anode generator (Rigaku UltraX-18HF) with monochromatic optics. The scattered radiation was detected in transmission mode by a mar345 image plate positioned 120 mm behind the samples. The scattering angles were calibrated with an α-alumina standard, and scattering intensities were corrected for absorption in the radiation path (Driemeier and Calligaris, 2011). The intensity was averaged across the azimuthal angles of the image plate to prepare X-ray diffractograms for presentation.

### Data Analysis

Cell wall fractions were grouped into four fractions to facilitate the polysaccharide degradation analyses during field exposure. The first group gathers sodium chlorite and ammonium oxalate data in pectin-rich and some soluble hemicellulose fractions. This fraction is named pectin-rich. The second group gathered 0.1 M and 1 M NaOH, which contains hemicelluloses less attached to cellulose fraction. It is called Hemicellulose A. The third group comprises hemicelluloses attached to cellulose fraction (4 M NaOH – named Hemicellulose B). The fourth group is the residue that consists of cellulose. Statistical analysis was performed with R software (version 3.4.1 – Copyright^©^ 2017 The R Foundation for Statistical Computing) by ANOVA one-way with posthoc Tukey-Kramer HSD (*p* < 0.05).

Principal component analyses (PCA) were performed to give a comprehensive view of how degradation occurs in sugarcane straw aged in the field. The variables measured were: ash content, lignin, soil humidity, carbon, uronic acid, nitrogen and isotopes, non-structural carbohydrates, and cell wall monosaccharide composition and fractions (*n* = 5). The ANOVA one-way tested the synthetic variables to verify the significant differences during the harvested months (*P* < 0.05). These analyses were performed in Minitab-14.1 software.

## Results

### Field Conditions

**FIGURE 2:**
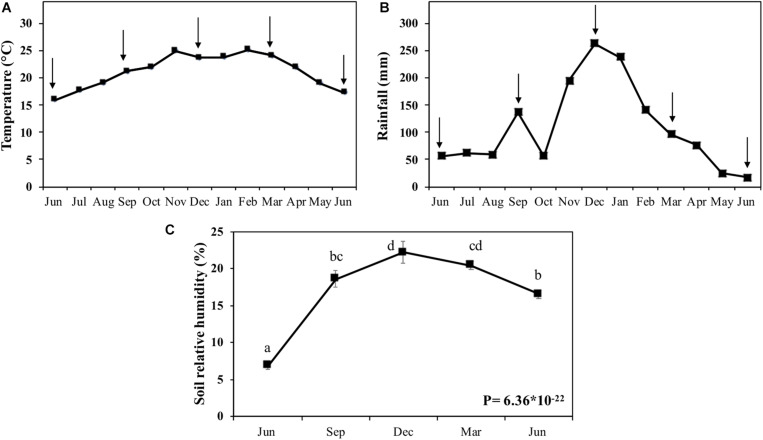
Climatic conditions and soil humidity in the field. **(A)** Average monthly temperature (°C). **(B)** Monthly rainfall (mm). **(C)** Soil relative humidity (%). **(A,B)** were generated from the data of Posto Agrometereológico do Departamento de Ciências Exatas da Escola Superior de Agricultura “Luiz de Queiroz,” Universidade de São Paulo (ESALQ/USP; http://www.esalq.usp.br/departamentos/leb/postoaut.html). Arrows indicate the harvest points that represent the filed times of 0, 3, 6, 9, and 12 months. Different letters are statistically distinct between the harvests in soil humidity (*P* > 0.05; *n* = 5).

Because the climatic conditions can influence the degradation process of biomass in the field, we measured temperature, rainfall, and soil humidity (Figure 2). The temperature varied by about 10°C from June 2009 to June 2010 (Figure 2A). The experiment started in the winter season, with an average temperature of 15°C. The temperature increased until November (25°C) and kept stable until March (spring to summer) when it decreased to 17°C in June of 2010 (winter).

Rainfall was less abundant (50 mm) during June-October 2009, except for September, when it peaked at 60 mm. From November to February of 2010, the rainfall increased, peaking in December with 263 mm (Figure 2B). From February of 2010, the rainfall decreased to reach 17 mm in June of 2010, when the winter started. The first layer of soil displayed humidity variation from 16.5–21.2%, generically following the rainfall pattern (Figure 2C).

### Non-Structural and Structural Carbohydrates Degradation

The non-structural carbohydrates (starch, glucose, fructose, sucrose, and raffinose) decreased 90% within 3 months on the field. Afterward, the level of sugars remained low in the plant tissues. The fructose and starch contents reduced less intensely than glucose and raffinose (78 and 88%, respectively) in the first 3 months (Table 1).

**TABLE 1:** Non-structural carbohydrates (starch, glucose, fructose, and raffinose) contents in the sugarcane aging 12 months in the field.

Time	Starch	Glucose	Fructose	Sucrose	Raffinose
0 months	16.97 ± 1.61^b^	8.48 ± 2.35^b^	7.74 ± 0.26^b^	37.30 ± 4.36^b^	0.95 ± 0.17^a^
3 months	2.01 ± 0.08^a^	0.22 ± 0.02^a^	1.67 ± 0.26^a^	0.51 ± 0.18^a^	0.00 ± 0.00^a^
6 months	2.86 ± 0.14^a^	0.21 ± 0.02^a^	1.63 ± 0.18^a^	0.25 ± 0.07^a^	0.00 ± 0.00^a^
9 months	2.62 ± 0.14^a^	0.25 ± 0.02^a^	1.69 ± 0.07^a^	0.35 ± 0.08^a^	0.00 ± 0.00^a^
12 months	1.57 ± 0.12^a^	0.21 ± 0.02^a^	1.65 ± 0.27^a^	0.34 ± 0.09^a^	0.00 ± 0.00^a^
*p*-value	**2.22 × 10^–9^**	**3.75 × 10^–5^**	**7.27 × 10^–5^**	**1.12 × 10^–10^**	**2.05 × 10^–5^**

*Values are represented by mean ± standard error expressed in μg⋅ mg^–1^ dry weight. Different letters are significant differences by Tukey’s test (*P* < 0.05; *n* = 5). Statistically significant *p*-values are shown in bold according to ANOVA one-way test.*

**FIGURE 3:**
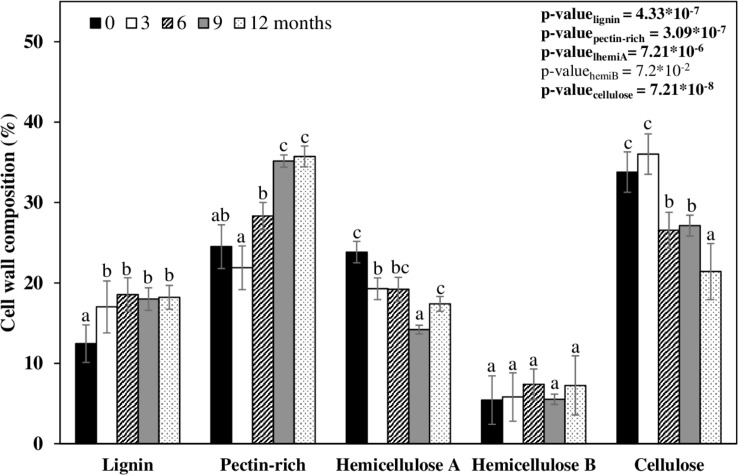
Relative percentages of cell wall composition of sugarcane straw for 12 months. Each bar represents the percentage of the component in the cell wall of each day sampled. A table with the values and statistics is in Supplementary Table 1 (*n* = 5).

The cell wall fractions are grouped into four categories – pectin-rich (sodium chlorite and ammonium oxalate fractions), hemicellulose A (0.1 and 1 M NaOH fraction), hemicellulose B (4 M NaOH fraction), and cellulose (treated residue; Figure 3 and Supplementary Table 3). Both the experimental design, and the data displayed in Figure 3, are intended to show the biomass composition on the day sampled, and not show how each cell wall fraction varied across time. The straw composition at month zero was 12.4% lignin, 24.5% pectin (some soluble hemicelluloses are also found but in minor proportions), 23.8% hemicellulose A, 5.4% hemicellulose B, and 33.8% cellulose (Figure 3). This cell wall composition was arbitrarily defined as standard for intact biomass. After exposure of the straw for 3 months in the field, the cell wall proportions did not change (Figure 3). At 6 months, the straw biomass increased 3.8% in pectin, with a concomitant reduction of 4.6% in hemicellulose A, and 7.2% cellulose (Figure 3). The straw left in the field after 9 and 12 months decreased the hemicellulose by 11.2% and the cellulose by 12.4% (Figure 3). In this period, the pectin-rich plus soluble hemicelluloses contents proportionally increased by 6.5% in comparison to the intact biomass (Figure 3). This result indicates that pectin was not consumed by most microorganisms present in the soil. Another evidence suggesting that pectins are not degraded is that the content of galactose, fucose, and rhamnose – monosaccharides typical of pectins – increased with time and reduced the other monosaccharides, except for galactose, which remained constant (Table 2). Also, the uronic acid content in the sodium chlorite and ammonium oxalate cell wall fractions increased proportionally as sugarcane straw aged (Table 2). The uronic acids still remained in the other cell wall fractions (∼ 90 μg⋅ mg^–1^). Mannan, a polymer that belongs to the hemicellulose class build-up from mannose chains, is also not degraded (see mannose levels in Table 2). However, arabinoxylan (arabinose and xylose), the main hemicellulose of sugarcane, seems to have been degraded by half over the first 3 months (Table 2 and Supplementary Figure 1). It was also possible to identify arabinoxylans as more soluble and less soluble types. The more soluble arabinoxylan was found in the fractions from sodium chlorite and ammonium oxalate, with a significant reduction within 12 months (Table 2). A less soluble arabinoxylan type (more attached to cellulose) was seen in the fractions of 0.1, 1, and 4 M, with a significant reduction (Table 2). This reduction possibly demonstrates the degradation, solubilization, and access of the polysaccharides to hydrolysis by soil microorganisms over the experimental period (Supplementary Figure 1).

**FIGURE 4:**
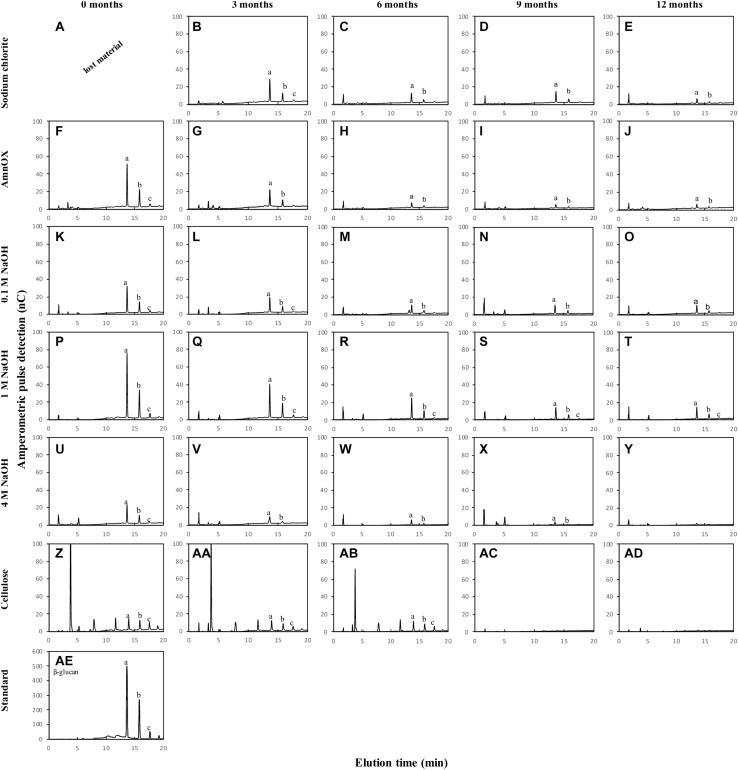
Oligosaccharide profile of cell wall fractions from sugarcane straw under field conditions for 12 months. The cell wall fraction was treated with a specific enzyme that cleave β-glucan - lichenase (Megazyme; *n* = 5). The cell wall fractions are represented on the y-axis and the straw field time in months on the *x*-axis. The β-glucan is determined by the peaks at the retention times of 13 and 16 min **(AE)**, therefore this pattern should be adopted for the evaluation of polysaccharide degradation on the fraction’s sodium chloride **(A–E)**, ammonium oxalate **(F–J)**, 0.1 M NaOH **(K–O)**, 1 M NaOH **(P–T)**, 4 M NaOH **(U–Y)**, and cellulose **(Z–AD)**. The lowercase letters inside the chromatogram represent **(a)** β-glucan trisaccharide, **(b)** β-glucan tetrasaccharide, and **(c)** β-glucan pentasaccharide. The data from **(A)** was lost during the sample processing (*n* = 5).

**TABLE 2:** Non-cellulosic monosaccharides (fucose, arabinose, galactose, rhamnose, glucose, xylose, and mannose) contents the sugarcane straw aging for 12 months on the field by TFA hydrolysis method and uronic acid content.

Fractions	Months	Arabinose	Fucose	Galactose	Glucose	Mannose	Rhamnose	Xylose	Uronic acids
AIR	0	26.89 ± 3.26^c^	0.12 ± 0.01^a^	5.88 ± 0.78^a^	23.46 ± 5.48^b^	0.83 ± 0.55^b^	0.00 ± 0.00a	89.33 ± 7.44^c^	–
	3	18.37 ± 1.12^b^	0.18 ± 0.03^ab^	4.91 ± 0.40^a^	9.59 ± 0.61^a^	2.52 ± 0.20^b^	0.54 ± 0.07b	73.62 ± 2.83^bc^	–
	6	14.63 ± 1.02^ab^	0.37 ± 0.03^bc^	5.02 ± 0.39^a^	10.83 ± 0.70^a^	3.41 ± 0.20^b^	0.67 ± 0.08b	60.54 ± 2.89^ab^	–
	9	10.43 ± 1.27^ab^	0.40 ± 0.07^c^	4.02 ± 0.69^a^	8.13 ± 1.49^a^	2.86 ± 0.50^b^	0.66 ± 0.12b	45.41 ± 3.34^a^	–
	12	11.41 ± 0.24^ab^	0.50 ± 0.08^c^	4.46 ± 0.19^a^	9.69 ± 0.56^a^	4.00 ± 0.34^b^	0.70 ± 0.06b	50.50 ± 1.71^a^	–
*p*-value		**0.000**	**0.000**	0.316	**0.003**	**0.000**	**0.000**	**0.000**	–
Sodium chlorite	0	31.69 ± 3.19^ab^	0.45 ± 0.02^a^	7.74 ± 0.25^a^	38.21 ± 6.32^c^	2.92 ± 1.31^a^	1.59 ± 0.13^a^	148.13 ± 22.70^a^	165.76 ± 2.96^ab^
	3	51.41 ± 1.30^c^	2.60 ± 0.51^ab^	8.12 ± 0.30^b^	17.57 ± 0.62^a^	2.50 ± 0.14^ab^	2.35 ± 0.12^ab^	207.11 ± 9.48^bc^	145.02 ± 11.29^a^
	6	38.04 ± 1.41^b^	1.42 ± 0.30^ab^	5.88 ± 0.23^b^	13.90 ± 0.40^ab^	3.41 ± 0.28^ab^	1.82 ± 0.10^ac^	153.27 ± 4.59^a^	179.83 ± 11.53^b^
	9	34.42 ± 1.04^ab^	0.96 ± 0.09^b^	5.88 ± 0.65^b^	14.91 ± 1.73^ab^	5.48 ± 0.97^b^	2.10 ± 0.10^c^	131.38 ± 3.41^a^	172.43 ± 3.3^ab^
	12	22.78 ± 5.37^a^	1.31 ± 0.21^b^	4.83 ± 0.48^b^	17.23 ± 0.64^bc^	3.54 ± 1.12^ab^	2.49 ± 0.20^bc^	94.44 ± 8.64^a^	165.94 ± 5.85^ab^
*p*-value		**0.000**	**0.005**	**0.002**	**0.002**	**0.011**	**0.002**	**0.000**	**0.061**
AmnOX	0	35.60 ± 3.61^cd^	1.47 ± 0.38^a^	11.95 ± 2.26^b^	71.21 ± 7.82^b^	7.17 ± 4.02^a^	1.75 ± 0.51^a^	106.03 ± 13.22^b^	118.91 ± 2.81^a^
	3	39.63 ± 2.17^*d*^	1.95 ± 0.10^b^	20.53 ± 1.19^b^	35.98 ± 2.14^a^	10.91 ± 0.69^a^	1.97 ± 0.11^bc^	191.86 ± 5.65^c^	143.72 ± 3.34^b^
	6	26.87 ± 1.26^bc^	2.63 ± 0.07^ab^	22.67 ± 0.57^a^	45.10 ± 1.43^a^	18.08 ± 0.45^a^	3.39 ± 0.10^ab^	132.35 ± 6.23^b^	122.43 ± 2.69^a^
	9	21.10 ± 1.18^ab^	3.31 ± 0.21^a^	22.39 ± 1.19^a^	51.70 ± 2.77^a^	24.20 ± 1.56^a^	3.87 ± 0.25^ac^	131.51 ± 2.39^ab^	119.28 ± 5.05^a^
	12	16.23 ± 3.34^a^	3.04 ± 0.59^a^	19.61 ± 2.63^a^	54.11 ± 3.58^a^	15.75 ± 5.48^a^	3.51 ± 0.64^c^	104.75 ± 6.88^a^	134.28 ± 5.66^ab^
*p*-value		**0.000**	**0.000**	**0.000**	**0.000**	0.205	**0.000**	**0.000**	**0.001**
0.1 M NaOH	0	76.55 ± 3.70^a^	2.75 ± 0.20^b^	10.79 ± 1.23^a^	15.26 ± 1.35^ab^	2.29 ± 0.35^a^	0.74 ± 0.12^a^	269.61 ± 4.86^a^	121.31 ± 7.31^a^
	3	73.82 ± 1.28^a^	3.22 ± 0.16^b^	15.64 ± 0.65^c^	10.93 ± 0.96^ab^	2.04 ± 0.51^a^	0.92 ± 0.10^b^	256.84 ± 4.19^a^	152.43 ± 11.65^a^
	6	63.49 ± 3.29^a^	2.91 ± 0.16^b^	11.56 ± 0.48^ab^	6.79 ± 4.17^a^	1.88 ± 0.20^a^	2.31 ± 0.12^b^	253.01 ± 8.34^a^	133.35 ± 12.99^a^
	9	193.48 ± 43.00^a^	3.23 ± 0.15^b^	14.60 ± 0.68^bc^	21.27 ± 0.08^bc^	2.41 ± 0.14^a^	2.34 ± 0.14^b^	219.38 ± 2.45^a^	124.09 ± 10.47^a^
	12	133.16 ± 79.86^a^	0.66 ± 0.21^a^	15.10 ± 0.23^c^	25.08 ± 1.70^c^	6.72 ± 0.45^b^	0.93 ± 0.02^a^	276.75 ± 31.38^a^	111.15 ± 13.17^a^
*p*-value		0.163	**0.000**	**0.000**	**0.000**	**0.000**	**0.000**	0.261	0.152
1 M NaOH	0	73.49 ± 1.69^b^	1.01 ± 0.07^ab^	14.94 ± 0.66^a^	39.02 ± 1.02^a^	0.00 ± 0.00^a^	1.18 ± 0.30^b^	404.65 ± 13.51^ab^	101.50 ± 2.78^ab^
	3	93.60 ± 9.08^b^	3.58 ± 0.46^b^	12.18 ± 1.64^a^	36.57 ± 2.64^a^	1.81 ± 0.75^a^	0.36 ± 0.06^ab^	662.98 ± 41.80^b^	125.20 ± 8.84^b^
	6	72.22 ± 8.73^ab^	2.80 ± 0.65^bc^	12.98 ± 1.61^a^	43.40 ± 8.85^a^	2.77 ± 1.02^ab^	0.67 ± 0.20^ab^	544.07 ± 61.33^ab^	102.80 ± 13.07^ab^
	9	62.98 ± 4.01^ab^	1.17 ± 0.64^ac^	13.73 ± 0.69^a^	47.81 ± 3.16^a^	5.52 ± 0.28^b^	0.72 ± 0.20^ab^	507.73 ± 37.85^ab^	81.87 ± 8.2^a^
	12	44.84 ± 9.02^a^	0.47 ± 0.22^c^	10.75 ± 1.30^a^	31.33 ± 5.04^a^	3.59 ± 0.37^ab^	0.29 ± 0.10^a^	346.94 ± 63.68^a^	85.57 ± 11.08^ab^
*p*-value		**0.004**	**0.003**	0.422	0.275	**0.002**	**0.004**	**0.006**	**0.031**
4 M NaOH	0	38.23 ± 10.06^b^	nd.	9.16 ± 1.52^a^	33.58 ± 4.31^ab^	5.79 ± 1.18^a^	0.56 ± 0.44^a^	257.84 ± 69.78^a^	111.50 ± 6.37^a^
	3	32.84 ± 2.48^ab^	nd.	6.26 ± 0.21^a^	25.96 ± 1.74^a^	4.11 ± 0.23^a^	0.00 ± 0.00^a^	325.25 ± 18.26^a^	97.61 ± 5.13^a^
	6	26.96 ± 4.30^ab^	nd.	7.62 ± 0.70^a^	31.80 ± 2.82^ab^	3.61 ± 0.17^a^	1.30 ± 0.40^ab^	237.82 ± 28.79^a^	106.87 ± 10.99^a^
	9	22.12 ± 4.38^ab^	nd.	8.23 ± 0.83^a^	33.78 ± 2.25^ab^	5.72 ± 0.79^a^	2.09 ± 0.47^b^	226.82 ± 49.53^a^	107.61 ± 3.25^a^
	12	10.43 ± 4.52^a^	nd.	7.39 ± 0.36^a^	41.57 ± 0.81^b^	4.94 ± 0.32^a^	0.84 ± 0.06^ab^	161.04 ± 10.05^a^	93.17 ± 3.27^a^
*p*-value		**0.027**		0.223	**0.010**	0.117	**0.004**	0.128	0.271
Residue	0	0.06 ± 0.05	nd.	2.06 ± 0.19^ab^	48.39 ± 12.95^a^	1.36 ± 0.35^a^	nd.	2.32 ± 0.43^a^	nd.
	3	0.04 ± 0.04	nd.	1.14 ± 0.46^a^	69.26 ± 5.09ab	1.04 ± 0.43^a^	nd.	1.68 ± 0.28^a^	nd.
	6	nd	nd.	1.07 ± 0.63^a^	68.40 ± 9.70ab	0.98 ± 0.57^a^	nd.	1.94 ± 0.18^a^	nd.
	9	0.40 ± 0.04	nd.	3.18 ± 0.36^b^	42.21 ± 2.59^a^	4.05 ± 1.80^a^	nd.	3.92 ± 1.21^a^	nd.
	12	0.95 ± 0.08	nd.	2.72 ± 0.05^a^	88.40 ± 8.93b	2.40 ± 0.08^a^	nd.	2.25 ± 0.50^a^	nd.
*p*-value				**0.020**	0.008	0.124		0.173	

*Values are represented by mean ± standard error expressed in μg⋅ mg^–1^ dry weight. Different letters are significant differences by Tukey’s test (*P* < 0.05; *n* = 5). AIR (alcohol insoluble residue) represents the integrate cell wall and AmnOX represents ammonium oxalate fraction. nd. stands for not detected. Statistically significant *p*-values are shown in bold according to ANOVA one-way test.*

Glucose levels in AIR reduced after 3 months, indicating β-glucan degradation. Due to this glucose reduction (Table 2 and Supplementary Figure 1), the β-glucan content and fine structure have been evaluated (Figure 4 and Table 3).

**TABLE 3:** Tri: tetrasaccharide ratios in β-glucan of sugarcane straw aging in the field for 12 months.

Time (months)	Chlorite	AmnOx	0.1 M	1 M	4 M	Cellulose
0	2.31 ± 0.12^ab^	2.26 ± 0.01^ab^	2.29 ± 0.02^b^	1.94 ± 0.22	2.02 ± 0.15^c^	1.14 ± 0.12^c^
3	2.28 ± 0.19^b^	2.27 ± 0.02^ab^	2.32 ± 0.01^ab^	2.24 ± 0.05	2.41 ± 0.03^bc^	1.28 ± 0.02^bc^
6	2.64 ± 0.01^ab^	2.42 ± 0.02^a^	2.40 ± 0.02^b^	2.38 ± 0.16	2.73 ± 0.05^ab^	1.17 ± 0.04^c^
9	2.76 ± 0.02^a^	2.42 ± 0.13^ab^	2.38 ± 0.04^ab^	2.18 ± 0.01	2.61 ± 0.16^a^	1.46 ± 0.04^ab^
12	2.68 ± 0.09^ab^	2.32 ± 0.06^b^	2.34 ± 0.02^ab^	2.19 ± 0.01	2.53 ± 0.05^ab^	1.63 ± 0.01^a^
*p*-value	**0.0142**	**0.0484**	**0.0270**	0.2076	**0.0001**	**0.0001**

*The rations refer to β-glucan in cell wall fractions (Chlorite, AmnOx, 0.1 M, 1 M, and 4 M of NaOH, and cellulose). Values are represented by average ± standard error (*n* = 5). Different letters are significant differences by Tukey’s test (*P* < 0.05). *p*-values statistically significants are shown in bold numbers according to ANOVA one-way test.*

Our results indicate that β-glucan is mostly associated with the wall’s more soluble fractions (sodium chlorite, ammonium oxalate, and 0.1 M NaOH – Table 2). The decay in β-glucan was followed by HPAEC detection of unique fragments obtained after specific enzyme degradation. The decrease is observed in the sodium chlorite (Figures 4B–E), ammonium oxalate (Figures 4F–J), 0.1 M NaOH (Figures 4K–O), and 1 M NaOH (Figures 4P–T) fractions. β-glucan easier degradation could be verified by the decrease of the height of the peaks *a, b*, and *c* of the chromatograms (Figures 4F–T). The reduction of this polysaccharide was observed in all fractions after 6 months compared to the intact biomass (month zero; Figure 4). A minor amount of β-glucan was also found in the 4 M NaOH and the cellulose fractions (Figures 4Z–AD). However, the last changes are not quantitatively significant since no glucose changes can be seen in Table 2 for these fractions.

Slight changes in β-glucan fine structure have been observed (Table 3). In general, an increase in the trisaccharide – with proportionally higher β-1,3 linkages in comparison with the tetrasaccharide – denotes that more β-1,4 linked polymers exist progressively with aging in the field. We found a small proportion of β-glucan associated with cellulose, but its structure is completely different from the more soluble β-glucan polymer, displaying much more β-1,4 linked glucoses.

**FIGURE 5:**
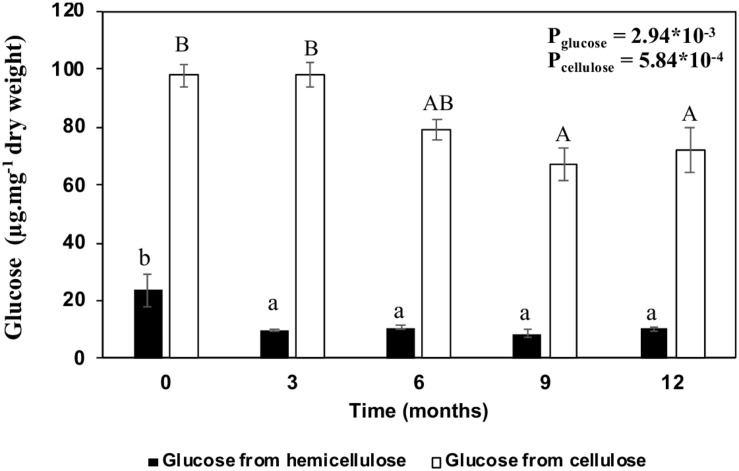
Comparison between glucose degradation from hemicellulose and cellulose of sugarcane straw in the field. Bars represent the average ± standard error. Dark bars represent the glucose from hemicellulose and light bars glucose from cellulose. Different letters are significant differences by Tukey’s test (*P* < 0.05; *n* = 5).

In summary, β-glucan degradation occurs in the first 3 months, with a glucose reduction of 57.9% compared to the initial stage (Figure 5). On the other hand, cellulose seems to take longer to be degraded. A reduction of 19% was observed after 6 months, reaching 26.2% after 12 months (Figure 5).

### Cellulose Crystallinity

**FIGURE 6:**
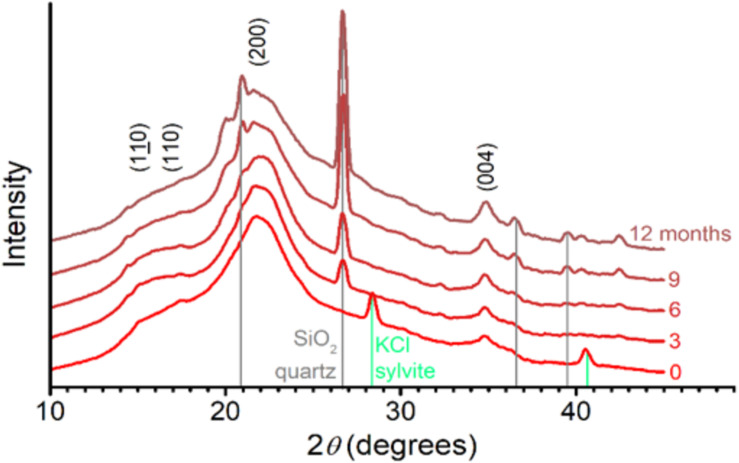
X-ray diffraction patterns of sugarcane straw aged in the field from 0 to 12 months. The main diffraction lines from crystalline cellulose (110, 110, 200, and 004) and contaminants – quartz (gray) and sylvite (green) – are indicated. Intensity is normalized and shifted for better visualization.

Diffraction patterns of sugarcane straw (Figure 6) showed the characteristic peaks of native crystalline cellulose (French, 2014), whichever the aging (0–12 months) on the field. This result demonstrated that native crystalline cellulose remained present in the residual biomass, although cellulose was partly degraded (Figures 3–5 and Table 2). It is noteworthy that a few diffraction peaks appear to be altered as the aging advances. In particular, the (004) peak became sharper and better defined, whereas the pair of peaks (110) and (110) became less defined. Based on the experimental data, it was impossible to conclude what type of slight alterations in cellulose crystals would be responsible for the observed diffraction changes.

**FIGURE 7:**
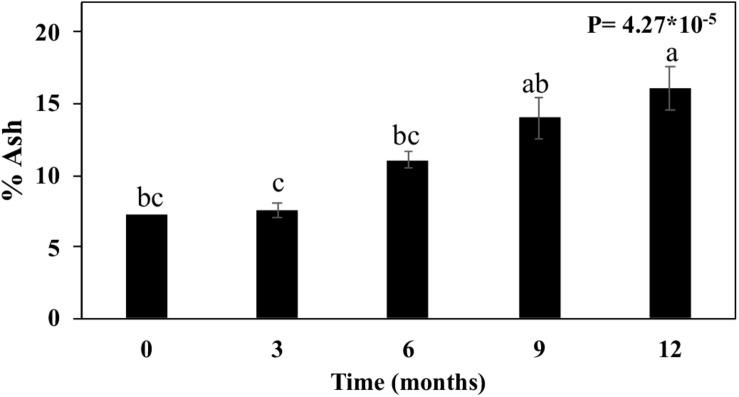
Ash content in sugarcane straw of different ages. Bars represent average ± standard error. Different letters are significant differences by Tukey’s test (*P* < 0.05; *n* = 5).

The diffraction patterns also showed a series of sharp diffraction peaks arising from mineral content (Figure 6). For the month zero, we observed peaks assignable to sylvite (KCl). Following the straw exposition in the field (3–12 months), the signal from sylvite disappeared, and diffraction peaks assigned to quartz (SiO_2_) became progressively more prominent (Figure 6). This mineral presence was also supported by increasing ash contents measured in straw (Figure 7). The ash content increased after 3 months by 3.9%, 6 months by 52.4%, 9 months by 92.8%, and 12 months by 121.4% (Figure 7).

### Biomass Recalcitrance and Its Impact on Saccharification

**FIGURE 8:**
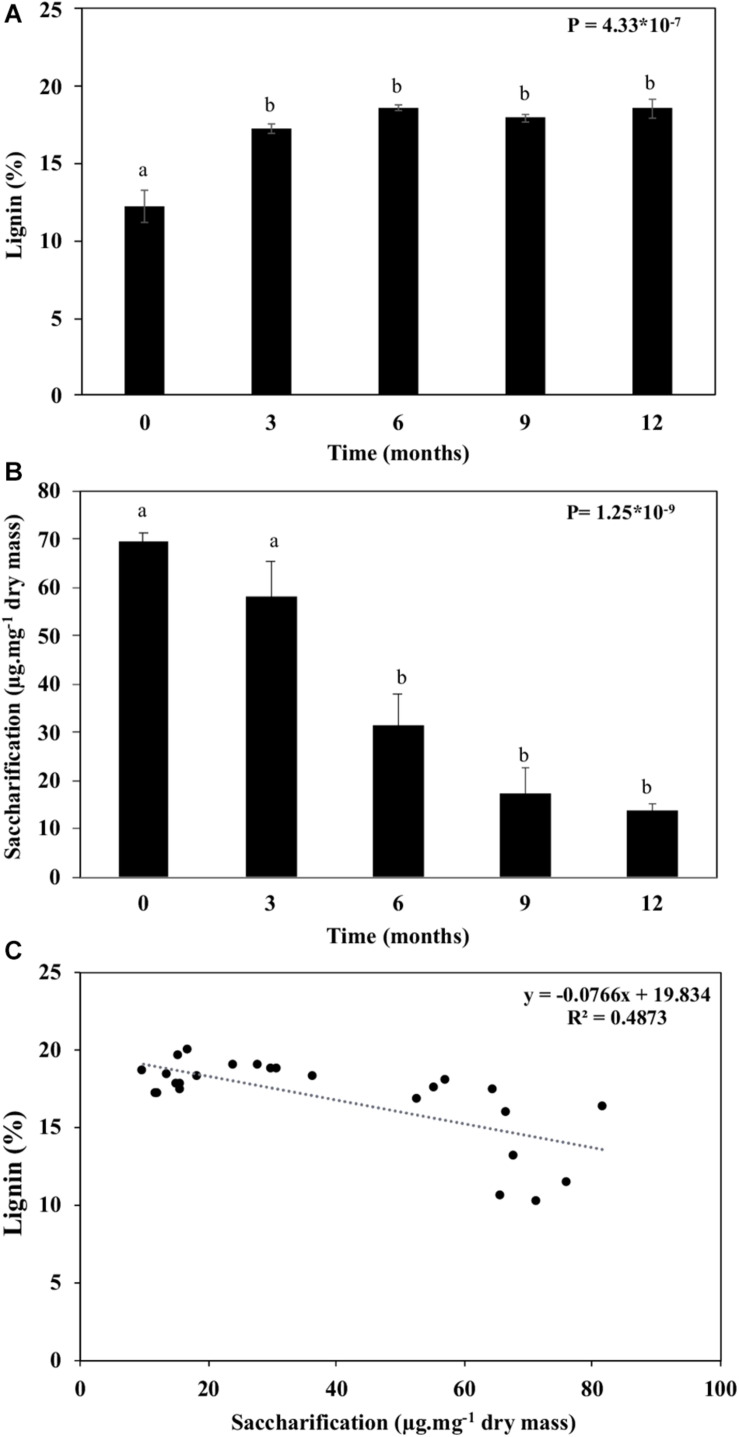
Lignin and saccharification of the sugarcane straw maintained under field conditions for 12 months. **(A)** Lignin quantification. **(B)** Total cell wall saccharification. **(C)** Correlation between lignin content and saccharification of the sugarcane straw. Bars represent the average ± standard error. Different letters are significant differences by Tukey’s test (*P* < 0.05; *n* = 5).

Lignin is a phenolic compound that cross-links with hemicelluloses and pectins within the wall. It is responsible for a significant proportion of the recalcitrance found in plant biomass, hindering saccharification processes. Structural and non-structural carbohydrates degradation along with proportional increases in cellulose crystallinity and lignin amounts (Tables 1, 2 and Figures 3–5, 8). The lignin increased proportionally to reducing structural and non-structural carbohydrates (Figure 8A). As a result, we observed a drastic decrease (80%) in the straw’s saccharification capacity with aging (Figure 8B). Saccharification and lignin displayed a negative correlation (Figure 8C), suggesting that lignin interferes in the cell wall access by the saccharification cocktail enzymes.

### Carbon and Nitrogen Variations

**FIGURE 9:**
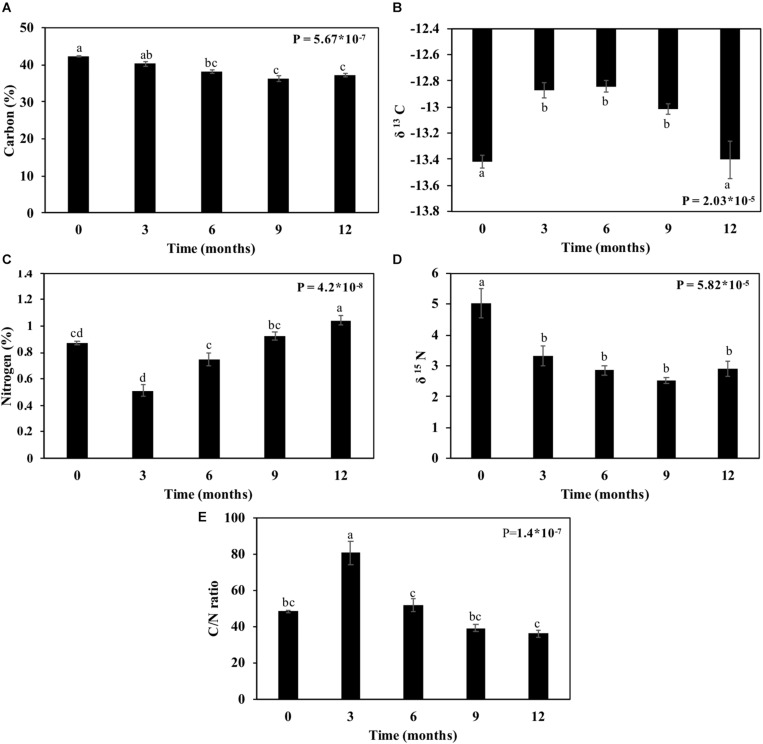
Carbon and nitrogen contents in sugarcane straw under field conditions for 12 months. **(A)** Percentage of carbon. **(B)** δ ^13^C ratio. **(C)** Percentage of nitrogen. **(D)** δ ^15^N ratio. **(E)** C/N ratio. Bars represent average ± standard error. Different letters are significant differences by Tukey’s test (*P* < 0.05; *n* = 5).

The straw’s carbon contents reduced after 12 months by 4.3%, and nitrogen content increased by 19.5% (Figures 9A,C). By evaluating these elements regarding their isotopes, the δ^13^C and δ^15^N decreased (Figures 9B,D). The reduction of the carbon content happened up to 9 months (4%), followed by an increase of 0.11% at 12 months (Figure 9B). The nitrogen content decreased significantly (34.2%) after 3 months and remained constant up to 12 months (Figure 9D). The C/N ratio increased during the first 3 months, peaking at around 80, and then reduced gradually up to 12 months to approximately 35 (Figure 9E).

### Principal Component Analysis

**FIGURE 10:**
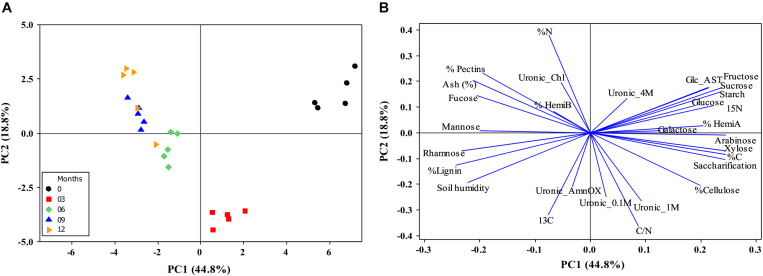
Distance biplots from straw on the field during 0 to 12 months. **(A)** The centroids separation corresponds to the straw harvest distribution for months harvests in the plane defined by the first and second main components (PC1 and PC2). Percentage values in parentheses (*x* and *y* axes) show the proportion of the variance explained by each axis. **(B)** Plot of the PC1 and PC2 loading vectors, describing the relationship among variables of straw composition during the harvests. The variables analyzed were expressed in descriptor vectors: Lignin, saccharification, ash, percentage and monosaccharide composition of cell walls (glucose, fucose, galactose, arabinose, xylose, rhamnose, and mannose), non-structural carbohydrates (glucose, fructose, sucrose, and starch), soil humidity, Uronic Acid, Carbon and Nitrogen (C and N), C/N ratio,15N, and 13C (*n* = 5).

The intact biomass (month zero) segregates from the naturally aged straw due to the starch, glucose, fructose, and sucrose levels. With aging in the field, the non-structural carbohydrates are degraded, the structural carbohydrates started to be modified, as hemicelullose A, and the lignin’s proportion on the biomass increased, decreasing the saccharification capacity. These separations of 0 and 3 months with the other months were explained by 44.8% data from PC1 (Figure 10). The increase of biomass recalcitrance is more evident after 6 months, the predominance of β-1,3 linkages from β-glucans suggests more accessible polysaccharides in the biomass but higher recalcitrance due to the high levels of pentoses, lignin, and some pectin (Tables 2, 3 and Figures 3, 8). Also, from 6 months onward, some cellulose degradation could be observed (Figure 5) with concomitant discrete changes in cellulose diffraction patterns (Figure 6), negative glucose vector (Figure 10), and an increase of minerals (PCA see positive Ash vector; Figures 7, 10). The carbon contents were reduced and concomitant to nitrogen increases after the first 6 months, possibly due to microorganisms’ action (Figures 9, 10), followed by a nitrogen intake after the rainy season started (Figure 2). The segregation of 0, 6, 9, and 12 months straw can be explained by PC2 (18.8%) due to high uronic acids contents in all cell wall fraction, C/N ratio, and δ^13^C, in 3 months, inversely to the increase N (Figure 10) in other months.

## Discussion

Sugarcane straw removal is thought to be related to soil quality (Castioni et al., 2018; Pimentel et al., 2019). Straw mineralization is thought to depend on biotic and abiotic factors, such as temperature and humidity, and chemical composition (Vitti et al., 2008). Thus, the sugarcane straw layer left on the field after the harvest and its decomposition are positively correlated to the soil dynamics. The decomposition of the straw left on the field is faster in the first months (Jensen et al., 2005; Fortes et al., 2012; Sousa et al., 2017), being sugars and proteins the first compounds to be degraded naturally (Coûteaux et al., 1995; Abiven et al., 2005; Sousa et al., 2017). This corroborates our observation of consumption of 90% of non-structural carbohydrates in the first 3 months (Table 1 and Figure 10). However, recalcitrance-associated compounds such as fats, tannins, and lignin are maintained on the material (Coûteaux et al., 1995; Abiven et al., 2005; Sousa et al., 2017). In our experiment, the absence of lignin degradation along with sugar consumption during aging in the field led to an increase of up to 5.8% of the lignin proportion in the biomass (Figures 3, 8A).

The environmental conditions and the microorganism’s biota influence the rate and degradation process (Rachid et al., 2016; Morais et al., 2019). The microorganism’s biodiversity is closely correlated to the soil origin, microfauna, nutrient availability, latitude, moisture, aeration, evapotranspiration, and temperature (Thorburn et al., 2003; Robertson and Thorburn, 2007). The temperature, rainfall, and consequently, the soil humidity can enhance microbial activity in soil and straw (Sousa et al., 2017). We observed that the peak of rainfall in the sugarcane field occurred between November (2009) and February (2010; Figure 2B). During this period, the soil humidity was kept at around 20% (Figure 2C), increasing the microbial activity and intensifying the plant cell wall biomass’s degradation between 6 and 9 months of aging.

The microorganisms that degrade plant tissues are fungi (e.g., *Aspergillus, Trichoderma*, and *Penicillium*) and bacteria (e.g., *Zymomnas* and *Cellulomonas*; Fortes et al., 2012; Valencia and Chambergo, 2013). *Trichoderma* and *Penicillium* have been shown to efficiently produce the complete set of enzymes capable to hydrolyze sugarcane biomass (Borin et al., 2015). These enzymes have to pass by the first cell wall barrier – the pectin, then hemicelluloses, and lignin to attack the cellulose. Thus, polygalacturonases (the main enzymes that attack pectins) are thought to be produced in the early stages to access the cell wall due to the depolymerization of homogalacturonans – the main pectin that is localized on the surface of the cell wall structure (de Souza et al., 2013; Bellincampi et al., 2014; Borin et al., 2015). Pectin is formed by the main chain of rhamnogalacturonan containing rhamnose and methyl esterified galacturonic acid that can be branched with chains of neutral sugars containing galactose and arabinose (Mohnen, 2008). The galacturonic acids are non-fermentable and harder to use as an energy source in metabolic routes. Therefore, despite the production of hydrolases, the microorganisms could be less efficient to use the main chain sugars for their energetic metabolism. We observed quite low pectin degradation in straw in the field, as seen by the persistence of galactose, fucose, rhamnose, and uronic acids (Table 2 and Figure 3). This corroborates the idea that, at least under the field conditions of our experiment, pectin degradation was not significant.

The susceptible hydrolysis of hemicellulose confers an easier and faster degradation process (Coûteaux et al., 1995). Considering hemicelluloses, A and B together, the reduction of the hemicelluloses content was 8.2% of the straw cell wall after 12 months (Figure 3). This finding is lower than the ones previously reported in the literature [Sousa et al. (2017; 23%), Oliveira et al. (1999; 21%), and Fortes et al. (2012; 33%)]. However, it is essential to emphasize that these studies used methods that do not fractionate the cell wall into all polymer classes (they only quantify cellulose and hemicelluloses), so that they may have probably computed pectins as hemicelluloses. Considering that 10% of the sugarcane walls are made of pectins (this work and de Souza et al., 2013), which were not degraded according to our observations, this explains the difference found between the present work and the literature.

The monosaccharides glucose, arabinose, and xylose were decreased by 41, 42, and 56%, respectively, denoting degradation of hemicelluloses such as β-glucan and arabinoxylans (see AIR in Table 2 and Figure 10). Arabinoxylan is the primary hemicellulosic polymer found in sugarcane, being a critical barrier to hydrolysis by microorganisms blocking the way to access cellulose (de Souza et al., 2013). Thus, xylanases and arabinofuranosidases are required for the microorganisms to access and hydrolyze cellulose (Grandis et al., 2019). In sugarcane, two types of arabinoxylans have been identified (de Souza et al., 2013). One is more soluble and appears to be the one that is degraded in the field. This is probably heavily acetylated and requires the action of acetyl esterases before xylanases can act (de Souza et al., 2013; Borin et al., 2015). The other seems to have remained practically intact during the 12 months of observation. It is likely that this would be the arabinoxylan that contains the branching with ferulic acid and hold lignin in the wall (dos Santos et al., 2008; de Souza et al., 2013, 2015). Another hemicellulosic sugar, the mannan, was not degraded, as denoted by an increase in mannose of 380% in the cell wall proportion (see mannose levels in Table 2). Although the mannan proportion is relatively small, it is an important polymer for recalcitrance, as it can strongly interact with other polymers, including cellulose.

Another polysaccharide that deserves attention is β-glucan. This polymer is classified as hemicellulose and contains glucose chains with mixed glycosidic linkages of the types β (1→3) and β (1→4). They are found in cereals (Buckeridge et al., 2004) and other grasses, including sugarcane (de Souza et al., 2013). β-glucan is thought to act as a scaffold for cell wall assembly in grasses (Buckeridge et al., 2004) and as a storage carbohydrate in seeds of cereals (Kiemle et al., 2014). In our observations, β-glucan is degraded with straw aging (Figure 4 and Table 3). The degradation was more evident after 6 months, and with aging, the polysaccharide became more soluble and less adhered to cellulose (Figure 4). With time, β-glucan increased the tri:tetra linkages ratios (Table 3), implying a predominance of β (1→3) linkages that confer higher solubility and consequently less rigid structure due to the weaker interaction with cellulose (Table 3). The modification in the pattern of β-glucan linkages suggests that fungi and bacteria might act more promptly on β (1→4) linkages than on β (1→3). Usually, β-glucans and cellulose are degraded by endo-1,4-β-glucanases, cellobiohydrolases, and β-glucosidases types of enzymes that break the β(1→4) linkage (Béguin and Aubert, 1994). Other enzymes might be present, such as lichenases (specific to β-glucan) and β (1→3)-glucanases (specific to callose; Borin et al., 2015; Grandis et al., 2019). Thus, the modification on the tri:tetra ratio potentially affects the degrading enzymes.

In the sugarcane cell wall degradation processes, after the pectin disruption and hemicellulose modification, cellulose becomes available for degradation (de Souza et al., 2013; Leite et al., 2017). In our observations in the field, the glucose content related to cellulose reduced by 26.7% at the end of the 12 months, reaching its peak at 9 months (Figure 5), which represents 12.4% of the cell wall degradation (Figures 3, 10). Sousa et al. (2017) and Fortes et al. (2012) estimated cellulose degradation as ∼10% biomass. Here, the native crystalline cellulose diffraction pattern was independent of the aging (0–12 months), although we have evidence that some changes in crystallinity and cellulose hydrolysis occurred (Figures 3, 5, 6). We demonstrated that biomass degradation changes the molecular environment in which the cellulose crystals are embedded, possibly promoting the relaxation relaxation of crystal stresses and changes to x-Ray diffraction peak widths (Figure 6). These modifications may be detectable for other polymers that increase in the straw during 12 months as an increase of pectins, lignin, and change in the hemicelluloses composition (Figure 3 and Tables 2, 3).

The diffraction patterns also show a series of sharp diffraction peaks arising from mineral content (Figure 6). The presence of sylvite (KCl) in the intact biomass could result from precipitation upon drying of the native K and Cl present as mobile nutrients in sugarcane leaves (Menandro et al., 2017). As the straw aged, the diffraction peaks assigned to quartz (SiO_2_) became more prominent. Quartz is a common mineral in soils, suggesting the impregnation of soil particles in the biomass structure. These minerals’ presence is also supported by the increasing ash content in straw after 3 months until 12 months when the percentage of ash increased by more than 100% (Figure 7). Notably, straw tissues were disrupted during field exposition, creating morphological irregularities, and opening the biomass’s intraparticle porous space. These morphological features ease soil debris trapping by the biomass structure (Negrão et al., 2019), is consistent with our observations.

As the proportion of pectin (Figure 3 and Table 2) and lignin arose on the total biomass (Figure 8A), due to the loss of other sugars (Tables 1, 2), the saccharification capacity of the biomass decreased by 80% within 12 months (Figure 8B). The most significant drop of the saccharification capacity corroborates with 90% of non-structural carbohydrates degradation (Table 1) along with the increase in the xylose:glucose ratio (Table 2). Therefore, for a higher yield of bioethanol, the straw harvest should not exceed 3 months in the field. After this period, fermentable sugars and cell wall polysaccharides will be lost, with a concomitant increase of biomass impurities (lignin, ash, and minerals; Tables 1, 2 and Figures 3–8, and 10). On the other hand, maintaining a thick layer of straw on the soil surface creates better environmental conditions for decomposing microorganisms, which speed up the carbon mineralization on the soil (Coppens et al., 2006; Curtin et al., 2008).

Carbon and nitrogen dynamics during the straw decomposition reveals the possible mineralization of the soil. The carbon from straw biomass was reduced with aging, and δ^13^C increased (Figures 9A,B). The lighter carbon (^12^C) was first consumed than heavy carbon (^13^C), as seen in some studies that show a higher content of the ^13^C on organic matter from the soil due to the preferential consumption of ^12^C (Blair et al., 1985; Natelhoffer and Fry, 1988; Mary et al., 1992; Schweizer et al., 1999; Fernandez et al., 2003; Mohagheghi et al., 2006). Also, the carbon is lost after the CO_2_ respiration of microorganisms on the sugarcane straw. As carbon was reduced, δ^15^N followed the same way (Figure 9D). The depletion of N can be explained by the reduction of uptake, leaching, and nitrification along with the bacterial biomass increase (Gautam et al., 2016). The %N increased, representing the N in plant biomass as reflecting from the total ecosystem N pool (Craine et al., 2015).

The carbon and nitrogen ratios can be used to gauge the state of mineralization and mobilization for crop intake (Brust, 2019). Usually, C:N of straw is above 50 (Brust, 2019) and decreases during the harvesting months, perhaps due to microbial degradation (Fortes et al., 2012). In the present work, the C:N ratio decreased over time, and after 12 months, its reduction was 36% (Figure 9E). The smaller the C:N ratio, the faster the mineralization. The result is the release of nitrogen, while the balance is given in a ratio between 20 and 30 (Watson et al., 2002). C:N ratios greater than 35, as is the case reported here (Figure 9E), may signify immobilization of microorganisms as mentioned by Watson et al. (2002). Therefore, the soil mineralization of straw maintained on the field will probably occur after 12 months when a C:N ratio becomes lower than 35. This will undoubtedly benefit soil recovery.

## Conclusion

As sugarcane straw aged in the field for a year, non-structural carbohydrates were degraded, the structural carbohydrates started to be modified, and the proportion of the lignin in the biomass increased, reducing saccharification capacity. We suggest that integrated harvesting is best for the sugarcane optimization harvest. The integrated harvesting use the straw harvest principle along with the sugarcane stalks, leaving behind only a small fraction of the straw for soil quality maintenance.

## Data Availability Statement

The raw data supporting the conclusions of this article will be made available by the authors, without undue reservation.

## Author Contributions

AG, AS, CS, and MB designed the experiment. AG, AS, and CS collected the plant material in the field. AS, CS, and DP performed the biochemical analyses. CD analyzed cellulose crystallinity and ash content. AG, AS, CD, CS, DP, and MB analyzed the data. AG, DP, and MB wrote the manuscript. All authors contributed to the article and approved the submitted version.

## Conflict of Interest

The authors declare that the research was conducted in the absence of any commercial or financial relationships that could be construed as a potential conflict of interest.

## Footnotes

1 http://www.esalq.usp.br/departamentos/leb/postoaut.html, accessed on October 10, 2018.

## Supplementary Material

Supplementary Figure 1 Distance biplots from significative monosaccharides from cell wall fractionation in straw on the field in the period of 1 year. **(A)** The centroids separation corresponds to the straw harvest distribution for months in the plane defined by the first and second main components (PC1 and PC2). **(B)** Plot of the PC1 and PC2 loading vectors, describing the relationship among variables of straw composition during the harvests. Percentage values in parentheses (*x* and *y* axes) show the proportion of the variance explained by each axis. The variables analyzed were expressed in descriptor vectors from significant (*P* < 0.05) monosaccharides in cell wall fractionation describe in Table 2. For statistics by PC1 and PC2 see Supplementary Table 3 (*n* = 5).

Supplementary Table 1 Relative percentage of cell wall composition statistic data. Values are represented by mean ± standard error. Different letters are significant differences by Tukey’s test (*P* < 0.05; *n* = 5).

Supplementary Table 2 Eigenvalues, proportions, and cumulative variance corresponding to each of the axes (PC1 and PC2) generated by the Principal Components Analysis (PCA) of the straw from five harvests, for 1 year. Values of the coefficients calculated for each of the variables measured along with the experiment of the of cell wall components (AIR Samples), non-structural carbohydrates, ash, saccharification, lignin, soil humidity, Uronic Acid, Carbon and Nitrogen (C and N), C/N ratio, ^15^N, and ^13^C parameters. ANOVA one-way test was performed to test the significance of the synthetic variables for each principal component (PC) and expressed in *F* and *P*-values. In bold the main vectors to represent the PC and bold/italic correspond to significant differences in PC for the time of harvest (0, 3, 6, 9, and 12 months; *n* = 5).

Supplementary Table 3 Eigenvalues, proportions, and cumulative variance corresponding to each of the axes (PC1 and PC2) generated by the Principal Components Analysis (PCA) of the straw from five harvests, for 1 year. Values of the coefficients calculated for each principal component in significative values (*P* < 0.05), from monosaccharide composition in a complete cell wall fractionation (Table 2). ANOVA one-way test was performed to test the significance of the synthetic variables for each principal component (PC) and expressed in *F* and *P*-values. In bold the main vectors to represent the PC and bold/italic correspond to significant differences in PC for the time of harvest (0, 3, 6, 9, and 12 months; *n* = 5).
