# Supplementary material for: The Effect of Sugarcane Straw Aging in the Field on Cell Wall Composition
Source: Front Plant Sci. 2021 Jul 15;12:652168. doi: 10.3389/fpls.2021.652168 (PMC8319731; doi:10.3389/fpls.2021.652168)
Supplement: Supplementary Figure 1 — Distance biplots from significative monosaccharides from cell wall fractionation in straw on the field in the period of 1 year. (A) The centroids separation corresponds to the straw harvest distribution for months in the plane defined by the first and second main components (PC1 and PC2). (B) Plot of the PC1 and PC2 loading vectors, describing the relationship among variables of straw composition during the harvests. Percentage values in parentheses (x and y axes) show the proportion of the variance explained by each axis. The variables analyzed were expressed in descriptor vectors from significant (P < 0.05) monosaccharides in cell wall fractionation describe in Table 2. For statistics by PC1 and PC2 see Supplementary Table 3 (n = 5). [file Data_Sheet_1.docx]

Supplementary Material

**Supplementary Table 1**. Relative percentage of cell wall composition statistic data. Values are represented by mean ± standard error. Different letters are significant differences by Tukey´s test (P<0.05) (n=5).

|  | **Lignin** | | | **Pectin** | | | **Hemicellulose A** | | | **Hemicellulose B** | | | **Cellulose** | | |
| --- | --- | --- | --- | --- | --- | --- | --- | --- | --- | --- | --- | --- | --- | --- | --- |
| 0 months | 10.92 | ± | 0.29^a^ | 24.79 | ± | 3.23^a^ | 23.58 | ± | 2.27^d^ | 6.02 | ± | 1.32ª | 34.66 | ± | 1.25^c^ |
| 3 months | 10.88 | ± | 1.57^a^ | 24.37 | ± | 2.72^a^ | 20.12 | ± | 1.53^cd^ | 6.14 | ± | 0.86^a^ | 38.46 | ± | 1.34^c^ |
| 6 months | 19.38 | ± | 2.78^b^ | 27.57 | ± | 1.34^ab^ | 19.46 | ± | 1.86^bc^ | 7.16 | ± | 0.64^a^ | 26.40 | ± | 1.32^b^ |
| 9 months | 23.08 | ± | 2.29^bc^ | 32.40 | ± | 3.01^b^ | 13.11 | ± | 1.84^a^ | 5.44 | ± | 0.70^a^ | 25.95 | ± | 3.67^a^ |
| 12 months | 27.24 | ± | 3.32^c^ | 31.28 | ± | 2.51^b^ | 15.97 | ± | 2.17^ab^ | 6.22 | ± | 1.29^a^ | 19.27 | ± | 2.98^a^ |
| **p-value** | **3.82*10^-10^** | | | **1.72*10^-4^** | | | **5.87*10^-7^** | | | 0.154 | | | **2.45*10^-10^** | | |

**Supplementary Table 2.** Eigenvalues, proportions, and cumulative variance corresponding to each of the axes (PC1 and PC2) generated by the Principal Components Analysis (PCA) of the straw from five harvests, for one year. Values of the coefficients calculated for each of the variables measured along with the experiment of the of cell wall components (AIR Samples), non-structural carbohydrates, ashes, saccharification, lignin, soil humidity, Carbon and Nitrogen (C and N), C/N ratio, ^15^N, and ^13^C parameters. ANOVA one-way test was performed to test the significance of the synthetic variables for each principal component (PC) and expressed in F and P-values. In bold the main vectors to represent the PC and bold/italic correspond to significant differences in PC for the time of harvest (0, 3, 6, 9, and 12 months) (n = 5).

**Supplementary Table 3.** Eigenvalues, proportions, and cumulative variance corresponding to each of the axes (PC1 and PC2) generated by the Principal Components Analysis (PCA) of the straw from five harvests, for one year. Values of the coefficients calculated for each in significative values (P<0.05), from monosaccharide composition in a complete cell wall fractionation (Table 2). ANOVA one-way test was performed to test the significance of the synthetic variables for each principal component (PC) and expressed in F and P-values. In bold the main vectors to represent the PC and bold/italic correspond to significant differences in PC for the time of harvest (0, 3, 6, 9, and 12 months) (n = 5).

**Supplemental Figure 1.** Distance biplots from significative monosaccharides from cell wall fractionation in straw on the field in the period of one year. **A.** The centroids separation corresponds to the straw harvest distribution for months in the plane defined by the first and second main components (PC1 and PC2). **B.** Plot of the PC1 and PC2 loading vectors, describing the relationship among variables of straw composition during the harvests. Percentage values in parentheses (x and y axes) show the proportion of the variance explained by each axis. The variables analyzed were expressed in descriptor vectors from significant (P<0.05) monosaccharides in cell wall fractionation describe in Table 2. For statistics by PC1 and PC2 see Supplementary Table 3 (n=5).
